# Neurodevelopmental Outcomes in Children with Neonatal Parechovirus CNS Infections

**DOI:** 10.3390/pathogens14060600

**Published:** 2025-06-18

**Authors:** Anna Piwowarczyk, Julia Śladowska, Agata Lipiec, Ernest Kuchar, Elżbieta Stawicka

**Affiliations:** 1 Department of Paediatrics with Clinical Assessment Unit, Medical University of Warsaw, 02-091 Warsaw, Poland; ernest.kuchar@wum.edu.pl; 2 Faculty of Medicine, Medical University of Warsaw, 02-091 Warsaw, Poland; s082748@student.wum.edu.pl; 3 Clinic of Paediatric Neurology, Institute of Mother and Child, 01-211 Warsaw, Polandelzbieta.stawicka@imid.med.pl (E.S.)

**Keywords:** parechoviruses, encephalitis, infants, developmental outcome

## Abstract

Human parechoviruses, officially known as Parechovirus A (PeV-A), are more frequently reported as a significant cause of serious infections in newborns and young infants. We aimed to describe the clinical features and neurological outcomes of PeV-A encephalitis cases identified in Warsaw. Infants with suspected encephalitis were retrospectively identified in three hospitals in the summer of 2022. Cases of confirmed PeV-A infection had their comprehensive demographic, clinical, laboratory, imaging, and outcome data reviewed. The psychomotor development of the children up to the age of 2 years was assessed by using the standardized tools. We identified 18 cases of confirmed encephalitis with a PeV-A infection. Their median age was 16 days. Fourteen cases were included in the analysis, while one patient dropped out after the first visit. Most were boys (9/14), and one patient was born preterm. Three patients had white matter alterations on brain MRI at discharge. No significant neurologic sequelae were observed after acute illness. At the 24-month follow-up, based on the Bayley Scales of Infant and Toddler Development (BSID-IV) and the Brunet–Lézine Scale, the children showed no neurodevelopmental sequelae. Brain MRIs were obtained in all of the participants up to 12 months of age and revealed no significant lesions. Neurodevelopmental complications are not frequent in children after PeV-A encephalitis at 24 months of age. Continued follow-up in larger cohorts is needed to explore the predictors of long-term morbidity.

## 1. Introduction

Human parechoviruses (PeV-A) are emerging as a significant cause of serious infections, including sepsis and central nervous system (CNS) infections with the need for pediatric intensive care unit (PICU) hospitalization, in young infants (<3 months of age) [1,2,3,4,5]. The neurological and developmental outcomes after early-life PeV-A encephalitis have not been systematically well-studied. The same data suggest a high frequency of severe neurological morbidity following PeV-A encephalitis [6,7,8]. In contrast, other studies report uneventful recovery in this group of children [9,10].

Human parechoviruses, reclassified from enteroviruses due to their distinct genetic and molecular features, comprise 16 serotypes, with PeV-A1–6 confirmed to cause human infections [11,12,13]. Among these, PeV-A3 is the primary neurotropic serotype associated with severe neonatal encephalitis, characterized by sepsis-like symptoms, periventricular white matter injuries on MRI, and long-term neurodevelopmental sequelae, including cerebral palsy [6,14,15,16,17,18,19]. While PeV-A1 and PeV-A2 are typically associated with mild gastrointestinal or respiratory illness, rare cases of encephalomyelitis (PeV-A1) and transient paralysis (PeV-A2) highlight their latent neuropathogenic potential [6,12,20].

The emergence of PeV-A3 as a dominant encephalitis pathogen reflects its unique epidemiological and biological traits. Neonates under three months of age are disproportionately affected due to their immature immune systems and the absence of maternal antibodies, with outbreaks following biennial patterns [6,17,19]. Unlike enteroviral encephalitis, PeV-A3 infections often lack cerebrospinal fluid (CSF) pleocytosis despite profound neurological injury, which complicates diagnosis [6,14,17]. Neuroimaging reveals distinctive diffuse white matter hyperintensity, correlating with motor and cognitive deficits [6,16,21]. A fourth serotype (K251176-02) has been identified in neonates with febrile illness, though its neurological implications remain undefined [12].

This serotype-specific pathogenicity highlights the need for PeV-A-aware diagnostic protocols, as standard enteroviral PCR is unable to detect PeV-A [12,16]. Understanding these associations is crucial for early intervention, given the absence of targeted antivirals and the reliance on supportive care or immunomodulators, such as IVIG [16,22,23].

The neurological outcomes following early-life PeV-A infections are still not fully understood, which could help with the implementation of effective prevention. However, early recognition might support prompt supportive management and prevent complications [24]. There are few studies summarizing the long-term neurodevelopmental outcomes, but there have been case reports of patients presenting with cerebral palsy, learning disabilities, or epilepsy after PeV-A encephalitis [6,8]. A recent meta-analysis suggests adverse neurological sequelae in up to 27% of cases in patients after a PeV-A neuroinfection [25]. However, some data suggest an increase in developmental manifestations later in life [25,26]. Further research is needed to verify the outcomes in longitudinal neurodevelopmental assessments of children with PeV-A-CNS infections.

The aim of this study was to investigate the long-term neurodevelopmental results in children after clinically and laboratory confirmed PeV-A encephalitis. We describe the key clinical features of this disease and its outcomes at discharge and 24-month follow-up to define severe CNS PeV-A infections better.

## 2. Materials and Methods

Subsequent to obtaining written informed parental consent, we retrospectively reviewed the medical records of infants younger than three months diagnosed with a suspected PeV-A CNS infection due to the 2022 infection outbreak. Children were hospitalized in three pediatric hospitals in Warsaw, Poland.

A case of PeV-A encephalitis was defined as any infant aged < 90 days presenting with a febrile illness or with other symptoms suggesting meningoencephalitis (an altered level of consciousness, lethargy, or behavior and/or personality changes) ≥24 h with ≥1 of the following symptoms—fever, seizures, focal neurologic findings, age-determined abnormal pleocytosis, or EEG/neuroimaging findings—and in whom PeV-A and no other pathogens were detected in the CSF [27]. CSF pleocytosis was defined as > 20 CSF leukocyte cells/uL in neonates, more than 9 cells/uL in infants aged 1 to 2 months, and > 5 cells/uL in children older than 2 months [28]. Additionally, we defined the sepsis-like course of a CNS PeV-A-infection based on the criteria for sepsis defined by the National Institute for Health and Care Excellence (NICE) [29]. Inflammation of the central nervous system was confirmed through positive rapid multiplex polymerase chain reaction (PCR) results from the CSF for common viral, bacterial, and yeast pathogens (FilmArray Meningitis/ Encephalitis Panel, BioFire Diagnostics, LLC, Salt Lake City, Utah 84108, USA). Positive PeV-A PCR results were not sequenced further for genotyping of the viral agents. The exclusion criteria included bacterial or viral co-infection and genetic disorders that could contribute to developmental delay.

### Procedures

The medical records of the infants were analyzed for their perinatal data and family history, clinical examinations, the course of the disease, the results of laboratory tests, and neuroimaging.

We assessed the neurodevelopmental outcomes prospectively in included patients using the BSID-IV [30] and the Brunet–Lézine scale [31]. The BSID-IV is an extensive formal developmental assessment tool for diagnosing developmental delays in early childhood and includes assessor-administered Language and Motor scale scores, in addition to the Cognitive scale. The Motor scale evaluates both gross and fine motor functioning. The BSID-IV is used for the age group starting from 16 days to 42 months of age, and scores are age-standardized. The Brunet–Lézine scale is used to assess the psychomotor development of children from 1 month to 30 months of age. It covers 4 aspects of psychomotor development: locomotion and postural control, eye-hand coordination, speech, and social skills.

Developmental assessments were conducted in hospital clinics every 3 months (at around the 3rd, 6th, 9th, 12th, 15th, 18th, 21st, and 24th months of life) by experienced neurologists (E.S., A.L.). During follow-up visits, a thorough history from the parents was recorded, and a neurological examination took place.

At the age of 12 months, a comprehensive neuropsychological evaluation was performed.

In the first year of life (at the age of 9–12 months), all patients underwent a brain MRI scan. MRIs were performed using 1.5 T imaging under general anesthesia in 13 patients.

## 3. Results

Among the 18 children identified to have confirmed PeV-A encephalitis during the 2022 outbreak, 14 parents consented to be contacted for future neurodevelopmental assessments. One patient did not complete the assessment, and in one case, the admission note could not be obtained.

### 3.1. Clinical Course and Results on First Hospitalization

Among the patients who completed the assessment (n = 13), nine were male and four were female. For those with available admission data (n = 12), the median age was 16 days (range: 8–58 days); one was born preterm, and one was an only child.

On admission, all patients presented with a subfebrile condition (n = 1) or the acute onset of a fever > 38 Celsius degrees (n = 11). Symptoms suggestive of central nervous system involvement included irritability (n = 11), decreased appetite (n = 5), hypertonia (n = 2), hypotonia (n = 2), vomiting (n = 1), and the presence of meningeal signs (n = 1). None of the infants demonstrated with seizures, cranial nerve involvement, or a bulging fontanelle. Four children were recognized to have a sepsis-like course of encephalitis. None of the patients demonstrated laboratory features of myocarditis. The median time of hospitalization was 7 days.

The CSF PCR tests were all positive exclusively for PeV-A (n = 12). A CSF analysis was not performed in one case due to diagnostic error. Among the 11 patients for whom CSF data were available, pleocytosis was observed in 3 cases, while elevated CSF protein levels were noted in 3 cases (N: <150 mg/dL and <58 mg/dL in infants younger and older than 10 days, respectively), and the glucose concentrations (N: >20 mg/dL) remained within the age-appropriate ranges in all patients.

During the acute phase of the disease, brain MRIs were performed in 8 children, transfontanelle ultrasounds in 10, and EEG in 2. The MRIs showed white matter alterations in the frontal lobes in three patients. Cranial ultrasound revealed a narrow longitudinal fissure in one case. All children who underwent EEG demonstrated the typical neurological patterns. None of the patients required PICU hospitalization. All patients were discharged without neurological sequelae.

### 3.2. Neurodevelopmental Assessments and MRI Scan Results During Follow-Up Visits

The study group included 14 patients, while 1 patient dropped out after the first visit. That patient, who was 4 months old at the time, had his psychomotor development assessed as typical. The first visit took place 2–4 months after hospital discharge, and the patients were between 3 and 6 months old at the time (average age: 4–6 months).

Thirteen patients had regular neurological follow-ups. They received comprehensive neurological, pediatric, and neuropsychological care according to an established regimen. As shown in the table, in the vast majority, no deviations were observed in the assessments of their psychomotor development. Only in one patient, with MRI abnormalities during her first hospitalization, was psychomotor development slightly delayed; despite motor rehabilitation, she reached the ability to sit at 11 months and to walk at 20 months. A delay in the development of expressive language was also observed in this child. The patient received the appropriate therapeutic interventions as part of an early intervention program.

On neurological examination, no major abnormalities were observed; among the minor neurological signs, a slight reduction in muscle tone and flaccidity were observed in four patients, while abnormal fluency of gaze and pseudostrabismus were seen in three patients. In two infants with pseudostrabismus, CSF pleocytosis was identified in the acute phase.

On CNS MRIs (at 9–12 months), features of incomplete myelination were described in six patients, and two patients had non-specific white matter lesions.

Table 1 shows the results after the completion of the neurodevelopmental assessments at the 12th and 24th months of age.

## 4. Discussion

We have presented a series of 14 consecutive infants who were prospectively observed in the areas of their motor, social, and communication skills after confirmed PeV-A encephalitis. Our results show that children at 2 years of age who had been hospitalized due to PeV-A CNS infections in early infancy presented developmental outcomes within the normal range according to the BSID-IV and the Brunet–Lézine scale. To our knowledge, this is the first study presenting brain MRI results after PeV-A encephalitis at 1 year of age of observation. All of our patients underwent a brain MRI 9–12 months after the acute phase of PeV-A encephalitis as a part of a comprehensive neurologic assessment. The abnormalities detected in the brain MRI scans in the form of incomplete myelination are considered to be a variant of the norm, related to the age of the patients at the time of the test (the patients were aged around 1 year). The normal process of CNS myelination progresses until the age of 2 years [32].

The literature is inconclusive about the long-term outcomes after a PeV-A CNS infection in early infancy. The overall favorable developmental outcomes of serious illness with PeV-A (e.g., sepsis-like or CNS infections) are consistent with reports from previous studies where no significant developmental impairments were found up to 24 months of follow-up [9,33,34,35]. On the other hand, the authors of the meta-analysis underlined an increasing average proportion of children who developed neurological or other developmental sequelae over time (5% at ≤6 weeks and 27% at ≥12 months) [25]. However, recent data on longer-term observations (>24 months of age) show an unfavourable impact on gross motor development after an enteroviral CNS infection at the age of 5 years, compared to control peers. There were no significant delays in earlier assessments. [35]. The authors linked worse long-term outcomes to PeV-A3 as the identified cause of the CNS infection in their cohort. PeV-A3 infection seems to have the strongest correlation with acute sepsis-like illness or encephalitis, and in vitro research has suggested that neutralizing antibodies may be less effective for PeV-A3 than PeV-A1 and that PeV-A3 may demonstrate a higher level of neurotropism [36]. In our cohort, we cannot draw definitive genotype-specific conclusions, as sequencing and determination of the type of parechovirus in the CSF are not the standard procedure in Poland and were not performed. Moreover, the recruited patients were retrospectively identified, which prevented this additional testing. However, based on previous reports, PeV-A3 is typically the most common type leading to encephalitis in infants [17,18,19,36].

One patient in an Italian cohort similarly showed a motor delay at the 48th month of age [37]. Moreover, results published by Briscoe et al. revealed a higher prevalence of Attention-Deficit/Hyperactivity Disorder (ADHD) in the PeV-A sample than that typical in the normal population, suggesting that PeV-A infections during infancy may be a risk factor for ADHD [38].

Importantly, children who experienced PeV-A encephalitis, in comparison to those without CNS involvement, have exhibited poorer outcomes [7,35]. The presence of neurological involvement, such as seizures or abnormal neuroimaging findings, may correlate with lower developmental scores and persistent neurocognitive deficits [6,32]. Developmental abnormalities were more often reported at follow-up in infants <3 months of age at the time of the initial infection in a large cohort of Australian children with PeV-A infection. They also presented with more serious and acute symptoms and were more frequently hospitalized in the PICU. A higher proportion of these infants were neonates or premature infants, which suggests that these factors may be not only associated with more severe acute infections but also a higher risk of subsequent developmental delay [7,25]. In addition, the age-related immature immune system and insufficient protective maternal antibodies may play a role in the vulnerability of the youngest children to the invasion of parechovirus into the CNS [39]. Data show that most children with neurological sequelae after PeV-A CNS infection (72.7%) required invasive ventilatory support and treatment with inotropes. Direct viral invasion, with secondary hemodynamic or respiratory consequences resulting in neurodevelopmental impairments could be the explanation for this outcome [5,7].

In the majority of studies, PeV-A CNS infections are associated with a young age and the absence of CSF pleocytosis [6,14,17,34,40,41], like in our cohort. While many infants have benign outcomes after CNS infections, there have been isolated reports of adverse neurodevelopmental outcomes in the context of abnormal neuroimaging [5,7]. Further studies with rigorous case definitions of PeV-A CNS syndromes are needed to understand the spectrum of PeV-A CNS infections better.

The limited sample size reduces the statistical power and generalizability of our findings. The retrospective process of identification for the patients recruited into the study made the observation of a bigger cohort of patients impossible. However, a prospective neurodevelopmental evaluation basing on the standardized protocol remains the strength of our study. Additionally, this study adds to the number of subjects followed up in this field.

Also, the lack of a control group presents as a potential limitation of our study. To lower the risk of bias, standardized scales appropriate for age, referring to the general population, were used for neurodevelopmental assessment. Moreover, previous research assessing the neurodevelopmental outcomes in children with parechovirus infections of the CNS in comparison to those in children with parechovirus infections elsewhere and with no pathogen detection shows that some controls also presented below the norm [33].

## 5. Conclusions

Summarizing the 2-year follow-up of these patients, in both the neurological examinations and the neuropsychological assessments, no significant deviations from normal psychomotor development were noted after PeV-A-CNS infections. However, because neurodevelopmental abnormalities in some domains might manifest or be recognized much later (e.g., ADHD), we recommend longer developmental monitoring of these children, preferably up to school age. Genotyping of PeV-A types could also support the treatment decisions and the knowledge concerning genotype-dependent outcomes and the need for early developmental interventions.

## Figures and Tables

**Table 1:** Long-term results of neurodevelopmental assessments.

Domain.	Percentage (N-Number)
MRI scan at the 9–12th month of age with incomplete myelination	46% (N = 6)
MRI scan at the 9–12th month age with non-specific white matter lesions	15% (2)
Proper neuropsychological assessment at the 12th month of age	92% (13)
Proper motor development at the 24th month of age	92% (13)
Proper speech development at the 24th month of age	92% (13)
Proper social development at the 24th month of age	100% (13)

## Data Availability

The raw data supporting the conclusions of this article can be made available by the authors on request.

## Abbreviations

The following abbreviations are used in this manuscript:

PeV-A	Human Parechovirus
MRI	Magnetic Resonance Imaging
BSID	Bayley Scales of Infant and Toddler Development
CNS	Central Nervous System
PICU	Pediatric Intensive Care Unit
CSF	Cerebrospinal Fluid
EEG	Electroencephalography
PCR	Polymerase Chain Reaction
ADHD	Attention-Deficit/Hyperactivity Disorder
